# Japanese Macaques (*Macaca fuscata*) as Natural Reservoir of *Bartonella quintana*

**DOI:** 10.3201/eid2112.150632

**Published:** 2015-12

**Authors:** Shingo Sato, Hidenori Kabeya, Aika Yoshino, Wataru Sekine, Kazuo Suzuki, Hidetoshi B. Tamate, Shouki Yamazaki, Bruno B. Chomel, Soichi Maruyama

**Affiliations:** Nihon University, Fujisawa, Japan (S. Sato, H. Kabeya, A. Yoshino, W. Sekine, S. Maruyama);; Hikiiwa Park Center, Tanabe, Japan (K. Suzuki);; Yamagata University, Yamagata, Japan (H.B. Tamate);; Japan Wildlife Research Center, Tokyo, Japan (S. Yamazki);; School of Veterinary Medicine, University of California, Davis, California, USA (B.B. Chomel)

**Keywords:** Bartonella quintana, bacteria, trench fever, wild macaque, Japanese macaques, Macaca fuscata, multilocus sequence typing, MLST, Japan, vector-borne infections, zoonoses

## Abstract

*Bartonella quintana* bacteremia was detected in 6 (13.3%) of 45 wild-caught Japanese macaques (*Macaca fuscata*). Multilocus sequence typing of the isolates revealed that Japanese macaques were infected with a new and specific *B. quintana* sequence type. Free-ranging Japanese macaques thus represent another natural reservoir of *B. quintana*.

*Bartonella quintana* is the causative agent of trench fever, which is characterized in humans by headache, recurrent fever, and pretibial pain. Major epidemics of the disease occurred among soldiers in Europe during World Wars I and II. More recently, trench fever has occurred sporadically in urban areas, mainly among homeless persons, drug-addicted persons, and HIV-positive patients in Europe and the United States (*1*). Body lice have been recognized as the only competent vector for *B. quintana* in humans, and poor hygienic conditions are strongly related to the occurrence of trench fever. Thus, *B. quintana* is considered a notable agent of a reemerging infectious disease.

Humans were thought to be the unique natural reservoir for *B. quintana* (*2*). However, this bacterium has also been isolated from cynomolgus macaques (*Macaca fascicularis*) bred in captivity in the United States (*3**,**4*) and from captive cynomolgus and rhesus macaques (*M. mulatta*) in China (*5**,**6*). These findings suggest that macaques may be another natural reservoir for *B. quintana*.

The number of wild Japanese macaques (*M. fuscata*) has recently increased throughout Japan, and these primates have become a serious nuisance by damaging crops, invading human residential areas, and biting persons (*7*). Because of this increasing human contact, if these primates become infected with *B. quintana*, they could transmit this bacterium to humans. However, no epidemiologic studies have been conducted to evaluate *B. quintana* in Japanese macaques and their role as a potential source of human *B. quintana* infection. Our goal was to investigate the prevalence of *B. quintana* in wild, free-ranging Japanese macaques and clarify the genetic characteristics of the strains by multilocus sequence typing (MLST).

## The Study

During July 2011–April 2014, a total of 45 blood samples were collected in EDTA-containing collection tubes from wild Japanese macaques in Aomori (n = 25), Yamagata (n = 5), and Wakayama (n = 15) Prefectures in Japan. The animals were captured by licensed trappers, in accordance with the Wildlife Protection and Proper Hunting Act, by using large hand-made cage traps and commercial cage traps (no. AM-181; Fujita Shoji Corp., Hiroshima, Japan). The physical conditions of each animal were recorded before they were euthanized, according to the guidelines of the Japanese Veterinary Medical Association. Freeze-thawed blood samples were spread onto chocolate agar plates (*8*) for isolation of *Bartonella* spp. and incubated at 35°C under 5% CO_2_ for up to 4 weeks. Then, CFUs per milliliter of blood were calculated. Five colonies from each culture-positive macaque sample were submitted for further characterization.

*Bartonella*-specific PCRs that targeted the *gltA* (*9*) and *rpoB* (*10*) genes and the 16S–23S rDNA intergenic transcribed spacer (ITS) regions (*11*) were used for identification of *Bartonella* isolates; genomic DNA of *B. alsatica* strain IBS 382^T^ and nuclease-free distilled water were used as positive and negative controls for the PCRs, respectively.

*Bartonella* isolates were obtained from 6 (13.3%) of 45 Japanese macaques; 1 (4.0%) of 25 macaques in Aomori, 1 (20.0%) of 5 in Yamagata, and 4 (26.7%) of 15 in Wakayama prefectures. No clinical signs were observed in the macaques with culture-positive samples. The bacteremia levels in the macaques ranged from 5.0 × 10^1^ to 3.7 × 10^4^ CFU/mL.

The DNA sequences of all 30 isolates were identical in the *gltA* (338 bp), *rpoB* (825 bp), and ITS (1,297 bp) regions; the sequences were registered in GenBank/European Nucleotide Archive in EMBL, and DDBJ under accession nos. LC031777 (*gltA*), LC031778 (*rpoB*), and LC031779 (ITS). BLAST searches (http://blast.ncbi.nlm.nih.gov/Blast.cgi) indicated that the DNA sequences of the isolates had the highest degree of similarity (100% for *gltA* and *rpoB*, 99.5% for ITS) with those of *B. quintana* RM11 strain from rhesus macaques. Subsequently, MLST analysis with 9 loci (*12*) revealed that 6 representative strains (MF1–1, MF3–1, MF10–1, MF11–1, MF19–1, and MF34–1 strains) from each culture-positive macaque were identical and belonged to a new sequence type (ST), ST22. The allelic profiles of ST22 and other STs are shown in the Technical Appendix Table.

**Figure 1:**
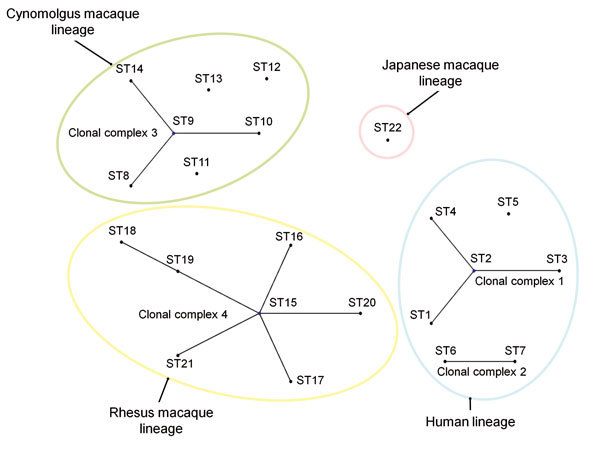
Phylogenetic relationship among 1 to 22 sequence types (STs) of *Bartonella quintana* strains based on eBURST analysis (http://eburst.mlst.net/default.asp). Black dots indicate ST numbers of *B. quintana* strains. A clonal complex was defined as a group of STs that had 8 identical alleles. Clonal complexes 1, 2, 3, and 4 consist of STs 1–4, STs 6–7, STs 8–10 and 14, and STs 15–21, respectively. A lineage was defined as a group of STs that had ≥7 identical alleles. Color circles show 4 lineages classified by host species.

A phylogenetic relationship between ST22 and other known STs was analyzed by using eBURST version 3 (http://eburst.mlst.net/default.asp) in combination with the MLST data. A clonal complex was defined as the group of STs that had identical alleles at 8 of 9 loci, and the lineage was defined as the group of STs that had identical alleles at 7 of the 9 loci. As previously reported (*6*,*12*), STs 1–4, STs 6 and 7, STs 8–10 and 14, and STs 15–21 formed clonal complexes 1, 2, 3, and 4, respectively, whereas ST22 remained a singleton (Figure 1). In terms of lineage classification, all STs, except ST22, were divided into 3 individual lineages by the host animal species: STs 1–7 for human strains, STs 8–14 for cynomolgus macaque strains, and STs 15–21 for rhesus macaque strains. In contrast, ST22 from Japanese macaque strains belonged to a singleton lineage.

**Figure 2:**
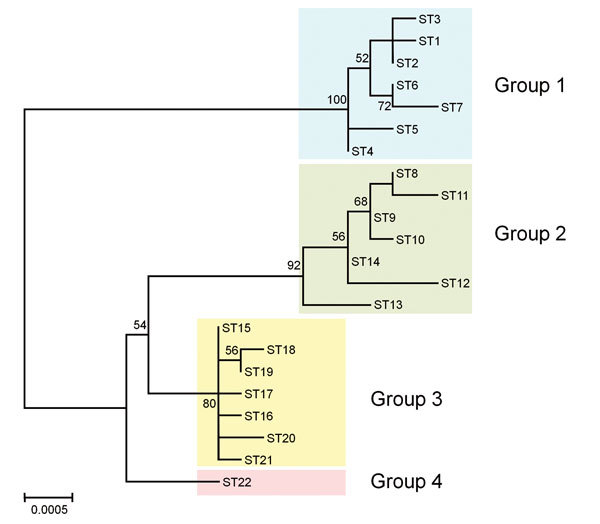
Phylogenetic tree showing the genetic relationship among *Bartonella quintana* strains from humans and macaques. The tree was constructed from the concatenated sequences (4,270 bp) of the 9 loci used for multilocus sequence typing by using the maximum-likelihood method based on the Tamura 3-parameter model in MEGA6 (*13*). The 22 sequence types (STs) of *B. quintana* strains from humans (STs 1–7), cynomolgus macaques (STs 8–4), rhesus macaques (STs 15–21), and Japanese macaques (ST22) were included in the tree. Colored rectangles show 4 groups classified by host species. The scale bar indicates estimated evolutionary distance. Bootstrap values were obtained with 1,000 replicates. Only bootstrap replicates >50% are noted.

We constructed a phylogenetic tree with the concatenated sequences (4,270 bp) of the 9 loci in each ST using the maximum-likelihood method in MEGA6 (*13*). The STs 1–7 from human strains, STs 8–14 from cynomolgus macaque strains, and STs 15–21 from rhesus macaque strains were classified into groups 1, 2, and 3, respectively, as with the lineage classification by eBURST analysis. All strains of ST22 formed a monophyletic clade defined as group 4 (Figure 2).

## Conclusions

Our study shows that natural infection with *B. quintana* can occur in free-ranging nonhuman primates in Japan. The Japanese macaques harboring *B. quintana* showed no clinical abnormalities, although bacteremia levels were considerably high (>10^3^ CFU/mL) in 3 animals that tested positive. These data suggest that Japanese macaques are one of the natural reservoirs of *B. quintana*. 

All isolates from Japanese macaques were more closely related to rhesus macaque strains than to human strains in the *gltA*, *rpoB*, and ITS regions. By MLST analysis with 9 loci, all 6 representative strains from Japanese macaques were identified as ST22 (*6*). Thus, ST22 is likely a new genotype of *B. quintana* specific to Japanese macaques. Because wild-caught Japanese macaques from only 3 prefectures were examined for *B. quintana* bacteremia, a large-scale surveillance study would help elucidate the genetic diversity of Japanese macaque strains.

According to eBURST analysis, human, cynomolgus macaque, and rhesus macaque lineages were formed by 4 clonal complexes. As reported previously (*6*,*12*), the primary founders in clonal complexes 1, 3, and 4 were reconfirmed as STs 2, 9, and 15, respectively. However, ST22 from wild-caught Japanese macaque strains was not found in any other clonal complex and formed an independent lineage. Through phylogenetic analysis with concatenated MLST sequences, Li et al. (*6*) showed that 3 groups were formed by each host species; this finding was confirmed in our study. However, ST22 from Japanese macaques formed another independent group (group 4). *Bartonella* spp. are known to have an adaptive strategy of causing asymptomatic and prolonged bacteremia in their specific reservoirs (*14*). Our data support the idea that *B. quintana* may have separately co-evolved with the macaque species and humans.

Notably, *Bartonella* DNA was recently detected in gorillas from West Africa, suggesting that nonhuman primates, including apes, could be naturally infected with *Bartonella* spp. (*15*). Further studies are necessary to clarify the prevalence of *B. quintana* and the vector of the organism in other nonhuman primates, and the potential of these primates to serve as a source of infection to humans.

Technical Appendix Allelic profiles and sequence types identified in *Bartonella qui*ntana strains.
