# Supplementary material for: Japanese Macaques (Macaca fuscata) as Natural Reservoir of Bartonella quintana
Source: Emerg Infect Dis. 2015 Dec;21(12):2168–70. doi: 10.3201/eid2112.150632 (PMC4672446; doi:10.3201/eid2112.150632)
Supplement: Technical Appendix — Allelic profiles and sequence types identified in Bartonella quintana strains. [file 15-0632-Techapp-s1.pdf]

Article DOI: http://dx.doi.org/10.3201/eid2112.150632

Japanese Macaques (*Macaca fuscata*) as Natural Reservoir of *Bartonella quintana*

Technical Appendix

Technical Appendix Table. Sequence types and allelic profiles identified in *Bartonella quintana* strains*

ST	Allele									Reference
	atpF	*bqtR*	*ftsZ*	*gap*	*gltA*	*groEL*	*nlpD*	*ribE*	*rpoB*	
1	1	1	1	1	1	1	1	1	1	*12*
2	1	1	1	1	1	1	1	1	2	*12*
3	1	1	1	1	1	1	2	1	2	*12*
4	1	1	1	1	1	2	1	1	2	*12*
5	1	1	1	1	1	2	2	1	3	*12*
6	1	1	2	1	1	3	1	1	2	*12*
7	2	1	2	1	1	3	1	1	2	*12*
8	3	2	3	2	2	4	3	2	4	*6*
9	3	2	3	2	2	5	3	2	4	*6*
10	3	3	3	2	2	5	3	2	4	*6*
11	4	1	3	2	2	4	3	2	4	*6*
12	3	1	4	2	3	5	3	2	4	*6*
13	3	1	5	2	2	6	3	2	4	*6*
14	3	1	3	2	2	5	3	2	4	*6*
15	1	1	4	3	4	6	4	3	4	*6*
16	1	1	4	3	4	6	4	3	5	*6*
17	1	1	4	3	4	6	5	3	4	*6*
18	3	1	4	4	4	6	4	3	4	*6*
19	1	1	4	4	4	6	4	3	4	*6*
20	1	1	4	3	4	6	4	3	6	*6*
21	1	1	4	3	4	6	4	4	4	*6*
22	5†	1	3	5	1	6	6	5	4	This study

*ST, sequence type.
